# Simultaneous Synthesis and Nitrogen Doping of Free-Standing Graphene Applying Microwave Plasma

**DOI:** 10.3390/ma13184213

**Published:** 2020-09-22

**Authors:** D. Tsyganov, N. Bundaleska, J. Henriques, E. Felizardo, A. Dias, M. Abrashev, J. Kissovski, A. M. Botelho do Rego, A. M. Ferraria, E. Tatarova

**Affiliations:** 1 Instituto de Plasmas e Fusão Nuclear, Instituto Superior Técnico, Universidade de Lisboa, 1049-001 Lisbon, Portugal; dtsyhanou@ipfn.ist.utl.pt (D.T.); julio.henriques@ist.utl.pt (J.H.); edgar.felizardo@ist.utl.pt (E.F.); ines.vieitas@tecnico.ulisboa.pt (A.D.); e.tatarova@ist.utl.pt (E.T.); 2 Faculty of Physics, Sofia University, 1164 Sofia, Bulgaria; mvabr@phys.uni-sofia.bg (M.A.); kissov@phys.uni-sofia.bg (J.K.); 3 BSIRG, iBB, DEQ, Instituto Superior Técnico, Universidade de Lisboa, 1049-001 Lisbon, Portugal; amrego@tecnico.ulisboa.pt (A.M.B.d.R.); ana.ferraria@tecnico.ulisboa.pt (A.M.F.)

**Keywords:** plasma synthesis, N-graphene, microwave plasma, atmospheric pressure

## Abstract

An experimental and theoretical investigation on microwave plasma-based synthesis of free-standing N-graphene, i.e., nitrogen-doped graphene, was further extended using ethanol and nitrogen gas as precursors. The in situ assembly of N-graphene is a single-step method, based on the introduction of N-containing precursor together with carbon precursor in the reactive microwave plasma environment at atmospheric pressure conditions. A previously developed theoretical model was updated to account for the new reactor geometry and the nitrogen precursor employed. The theoretical predictions of the model are in good agreement with all experimental data and assist in deeper understanding of the complicated physical and chemical process in microwave plasma. Optical Emission Spectroscopy was used to detect the emission of plasma-generated ‘‘building units’’ and to determine the gas temperature. The outlet gas was analyzed by Fourier-Transform Infrared Spectroscopy to detect the generated gaseous by-products. The synthesized N-graphene was characterized by Scanning Electron Microscopy, Raman, and X-ray photoelectron spectroscopies.

## 1. Introduction

The existing variety of carbon structures is due to the ability of the carbon atom to form different hybridizations, such as tetrahedral sp^3^-(diamond), sp^2^-trigonal (graphite, fullerene, nanotubes), or linear sp-(carbyne) [1]. Graphene is one of the examples, which has attracted great attention since it was first synthesized in 2004 [2]. The interest in graphene is dictated by its special structure (one atomic layer of sp^2^ carbon atoms bonded in a hexagonal configuration) and properties (high thermal and electron conductivity, mechanical strength, elasticity, high specific surface area, optical properties, etc.). These features arise from the unique nature of graphene’s charge carriers, which behave like relativistic particles. Thus, it is not surprising that graphene is seen as a promising material in nanoelectronics, “classical” microelectronics, as well as in spintronics, artificial neural networks, etc. [3,4,5]. It is believed that switching to carbon-based microelectronics, in particular graphene, could solve overheating issues, greatly reduce component size, improve responsiveness, etc. To accomplish this, it is necessary to manage the electronic structure of graphene. One approach is based on controlling the type of conductivity and carrier concentration, which can be achieved by doping with foreign atoms. Nitrogen is typically used as a doping agent, since it is easily incorporated in the graphene lattice, due to high reactivity and comparable atomic size. Nitrogen atoms can form different configurations in graphene, commonly pyridinic, pyrrolic, and graphitic, each of them affecting the electronic structure and transport properties of the functionalized graphene in a different way [6,7,8,9,10]. In the case of direct substitution of carbon with a nitrogen atom, i.e., graphitic N, the three valence electrons of the nitrogen atom participate in the formation of three σ bonds, the fourth electron occupies *π* state, and the fifth fills π* state of the conduction band of graphene, thus leading to n-type (metallic behavior) doping [11,12,13,14,15,16]. The addition of nitrogen atoms increases the electrochemical activity of the N-graphene due to formation of “active sites” on the graphene lattice. Thus, N-graphene is suitable as a catalyst or as a support matrix for the conventional catalysts [17,18,19].

Among various synthesis methods of graphene and graphene-related structures, plasma-based techniques have emerged as an eco-friendly and energy-saving alternative. For instance, one of the most common graphene synthesis methods, i.e., chemical vapor deposition (CVD), was significantly improved with the inclusion of plasma [20]. In the plasma-enhanced chemical vapor deposition (PECVD) technique, the plasma ensures the decomposition of the precursors and the temperature control of the substrate, as well as higher growth rates. A particular advantage of PECVD over classical CVD is the possibility of growing large nanostructures with vertically oriented architectures, which can be interesting for specific applications. Plasmas are successfully used to produce various nanostructures, such as nanorods, nanowires, nanocones, graphene, etc. [21].

N-graphene was successfully synthesized by nitrogen plasma treatment of graphene with regulated doping level, ranging from 0.11 to 1.35 at % N [22]. Radiofrequency (RF) ammonia plasma was used to dope graphene with 3 at % of N, demonstrating that longer plasma exposure leads to an increase of pyrrolic N in the structures [6]. Typically, a post-treatment plasma approach is applied, which employs N_2_ or ammonia plasma processing to previously synthesized graphene [10,22,23,24,25,26,27,28,29]. The nitrogen content can be controlled by tuning the plasma parameters and the exposure time, using different plasma sources. Graphene and highly oriented pyrolytic graphite (HOPG) were doped using RF nitrogen plasma with incorporated nitrogen in the range of 5–15 at % [24]. Doping levels as high as 25 at % of N can be reached using the post-treatment plasma approach, but with the doping occurring predominantly on the surface, i.e., not homogeneously. Moreover, such high doping levels lead to the formation of different carbon–nitride structures [29]. Applying the direct plasma approach can potentially provide homogeneous doping, since the synthesis and doping are simultaneously ongoing processes in the plasma environment. Additionally, free-standing N-graphene sheets, i.e., not attached to a substrate, are more suitable for some technological applications, since both surfaces can be used. Until recently, obtaining free-standing graphene or N-graphene sheets was considered impossible, since the thermodynamic analyses, computing the fluctuations in the positions of carbon atoms in the graphene lattice, indicated that free-standing 1D and 2D crystals are unstable [30,31]. However, these analyses were conducted under simplified assumptions, including the harmonic approximation and the formation of completely flat 2D crystalline structures. In practice, free-standing graphene/N-graphene sheets using atmospheric pressure microwave plasma reactor were already synthesized [32,33,34,35,36,37,38,39,40,41]. The synthesized substrate-free graphene sheets show both good mechanical and chemical stability, due to the curled nature of the structures. The microwave plasma-based technology offers several advantages in nanofabrication such as cost reduction and fast and scalable production in a controllable manner.

In this study, microwave plasma at atmospheric pressure was used for direct synthesis of free-standing N-graphene sheets. The work is a continuation of the research carried out within the framework of plasma-based synthesis of advanced 2D nanostructures, which includes graphene [32,33,34,35,37] and N-graphene [39,40] syntheses using various precursors. The synthesized nanostructures have been tested as low secondary yield coating materials [38] and as conductive matrix for Ni (OH)_2_-based supercapacitive electrodes [41]. In the present work, the feasibility of N_2_ gas as a graphene doping agent is experimentally and theoretically investigated. The theoretical studies were focused on revealing the main mechanisms of precursor’s decomposition and creation of the new species involved in the formation of N-graphene structures. For this purpose, the previously developed chemical kinetics model was updated with nitrogen-containing species and the corresponding rate coefficients.

## 2. Experimental Arrangements

**Figure 1:**
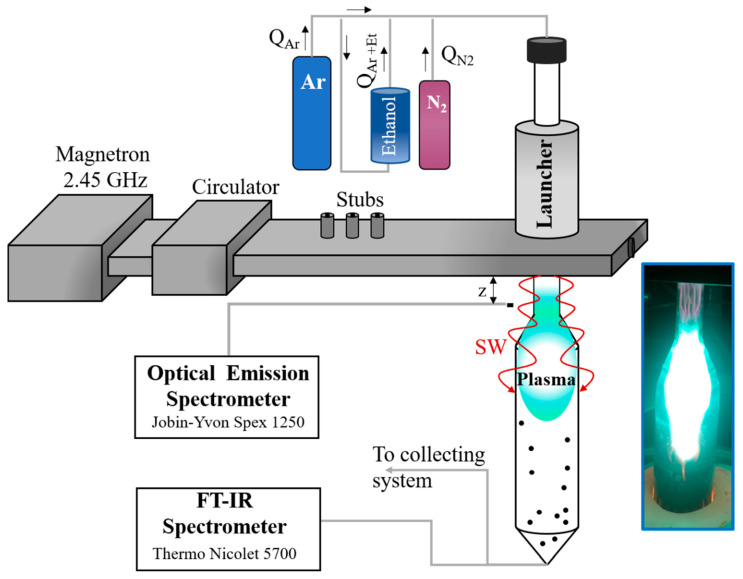
Scheme of the experimental setup.

A waveguide-surfatron-based setup was used to create a surface wave (SW)-induced microwave plasma at atmospheric pressure conditions [42]. SW-sustained discharges are produced by the field of a travelling wave that simultaneously propagates and creates its own propagation structure, creating an extended active zone outside the wave launcher (Figure 1). In this way, large microwave power densities can be injected into the processing area and high population densities of active species of interest can be achieved. Up to 2 kW of 2.45 GHz radiation is provided by a Sairem microwave generator connected to a waveguide-system (WR-340) composed of a water-cooled circulator, a three-stub tuner, a moveable short-circuit, and a waveguide-surfatron as a field applicator.

The discharge takes place inside a quartz tube inserted perpendicularly to the waveguide wider wall and directed downstream (Figure 1). Optimized reactor geometry in respect to our earlier works [32,33,34,35,37] includes a section of the quartz tube with expanding radius. The design permits control over the thermodynamic conditions (gas velocity, thermal fluxes, residence time etc.) of the plasma reactor. Argon (purity of 99.999%) is used as a background gas, while ethanol and N_2_ are the carbon and nitrogen precursors, respectively. The flow of the gas mixture injected into the plasma reactor and the applied microwave power (*p* = 2 kW) were kept constant. The ethanol was kept at a narrow range of flow rates which ensure graphene assembly. A set of experiments were performed varying the N_2_ gas flow (Q_N2_) injected into the plasma. The created nanostructures were captured by a tornado-like collecting system and accumulated in a glass container.

Optical Emission spectroscopy (OES) was applied to detect the emission of the plasma-generated “building units” and to determine the gas temperature. Optical fiber was placed perpendicularly to the discharge tube and directed to the entrance of a Jobin-YvonSpex 1250 spectrometer (1200/2400 g/mm grating) equipped with a charge-coupled device (CCD) camera. The cryogenic, back illuminated ultraviolet (UV)-sensitive CCD camera has a 2048 × 512 matrix, featuring a 13.5 µm pixel-size, which provides high spectral resolution. The plasma emission spectrum in the 230–800 nm range was detected.

A FT-IR Thermo Nicolet 5700 spectrometer was used to investigate the 1000–4000 cm^−1^ absorption spectra of species in the plasma outlet gas. The 2D map of the temperature at the reactor walls was monitored with thermal imager (FLIR systems).

Scanning Electron Microscopy (SEM) characterization of the samples was performed using a JEOL, JSM-7001F field emission gun scanning electron microscope operating in secondary electron imaging mode (SEI) using 15 kV of accelerating voltage. The samples were deposited on a double-sided carbon tape mounted on an aluminum stub.

Raman spectroscopy of the samples was carried out using a LabRAM HR Visible (Horiba Jobin-Yvon) Raman spectrometer with a spectral resolution of 1 cm^−1^ and a 633 nm He-Ne laser excitation with laser spot size of 2 μm. The synthesized nanostructures were freely suspended on a glass substrate and the Raman spectra from different regions on the substrate were obtained. Measurements were performed with a laser power *p* = 0.054 mW to avoid overheating.

N-graphene samples were also characterized by X-ray Photoelectron Spectroscopy (XPS) using a XSAM800 spectrometer from KRATOS Analytical. The incident radiation, Mg Kα X-rays with 1253.6 eV, was produced with a power of 120 W. Spectra were acquired in Fixed Analyzer Transmission (FAT) mode, with high magnification and a pass energy of 20 eV. Charge shift was corrected using as reference the binding energy 284.4 eV, assigned to sp^2^ carbon atoms in graphene [39]. Samples mounting and details on the data treatment were described in [39].

## 3. Theoretical Model

### 3.1. General Description

A previously developed theoretical model describing the chemical kinetics and the thermodynamics in the microwave plasma medium [35,40,43,44] was further updated in order to account for the formation of C, O, N-containing species. The algorithm is similar to that found on the commercial code Reaction Design, Chemkin (ANSYS, Inc.) [45]. The kinetic scheme is a combination of two mechanisms, i.e., the kinetic mechanism of ethanol decomposition (the hydrocarbon mechanism) based on the one developed by Marinov [46] and the full mechanism of formation of species containing C, O, N in flames (the so-called C/O/N mechanism) by Klaus and Warnatz [47,48]. The mechanism described in [46] does not consider carbon black formation, thus a carbon block containing new components (gas-phase C, C_2_) and around 20 new reactions from the kinetic mechanism of Konnov [49] and GRI [50] was added. Additionally, nitrogen-containing species, i.e., NH, HCN, N, CN^•^, NH_3_, NH_2_, NO_2_, HNO, NO, N_2_H, N_2_O, H_2_CN, HNO_2_, HCNO, NCO, HNCO, C_2_N_2_, and N_2_ are considered and the reaction mechanism with the corresponding rate coefficients is included. To find the most reliable values for the rate constants, extrapolation to the upper border of the experimentally determined temperature range (~3000 K) using a non-linear extrapolation method that solves the Fredholm integral equation was used [51]. The expressions for the rate constants in the extended temperature range were validated with data available in the literature [45,50,52,53,54,55]. The thermodynamic magnitudes for the kinetic scheme were taken from thermodynamic databases [45,50,52,53,54,55]. The updated kinetic scheme includes 85 species and about 537 chemical reactions [35,37]. The model output includes axial variations of the gas temperature and active species of interest in the “hot” and “mild” plasma zones. The contribution of the main “building blocks” to N-graphene formation is illuminated. It is assumed that the formation of the carbon solid phase is a diffusive mechanism. The rate of formation of C (solid) was estimated as [35,40]

$$k_{est}=a_{C/C_{2}}k_{0}\cdot \;{\left(\frac{T}{273}\right)}^{3/2}\approx a_{C/C_{2}}20\;{\left(\frac{T}{273}\right)}^{3/2}$$

Here the factor $a_{C/C_{2}}$ = 1 for C, and $a_{C/C_{2}}$ = 0.707 for C_2_. The estimated coefficient *k_est_* was tested by adopting the factor *k*_0_ as a free parameter. The best fit for *k*_0_ with the experimental results was found to be very close to the estimated diffusion rate coefficient of carbon species (*k*_0_ = 24), which supports the initial assumption of the diffusion nature of the carbon transport.

### 3.2. Basic Principles

The minimization of the Gibbs energy of the system was used in the equilibrium approach, while a set of differential equations is solved in the non-equilibrium approach. The chemical kinetic scheme was used to generate a set of differential equations, i.e., the system of non-linear mass balance equations and the total energy-conservation equation.

The mass balance is then represented by the set of equations:

$$\frac{dY_{i}}{dt}=\left(\omega_{i}-\xi_{i}\right)\frac{W_{i}}{\rho },i=1\ldots ns$$

where *i* is the species index; *Y_i_* is the mass fraction of species *i*; $\omega_{i}$ is the chemical molar production of species *i* (mol cm^−3^ s^−1^), $\xi_{i}$ is the chemical molar loss due to diffusion of species *i* (mol cm^−3^ s^−1^), $W_{i}$ is the molecular weight (g mol^−1^); ns is the total number of species; ρ is the total mass density (g cm^−3^) and *t* denotes the time (s).

The equation is solved in time until the steady state is reached. Additionally, a normalization condition accounting that the total mass fraction is given by $\sum_{i=1}^{ns}Y_{i}=1$ was imposed. The chemical source terms in the equations are calculated for the R elementary reversible or irreversible reactions involving *ns* chemical species: $\sum_{i=1}^{ns}\nu_{ir}^{\prime }A_{i}\overset{\;12\;}{↔}\sum_{i=1}^{ns}\nu_{ir}^{\prime \prime }A_{i},r=1\ldots R$.

Where $\nu_{ir}^{\prime }$ and $\nu_{ir}^{\prime \prime }$ are the stoichiometric coefficients of the forward and reverse reactions with $A_{i}$ species involved. The chemical production rate $\omega_{i}$ of the *i*-th species can be written as a summation of the rate-of-progress variables for reactions involving the *i*-th species, as shown in $\omega_{i}=\sum_{i=1}^{R\sum_{r}}\nu_{ir}^{\prime \prime }$.

The rate-of-progress variables $q_{r}$ for the *r*-th reaction are provided by the difference between the forward rates ‘1’ and the reverse rates ‘2’ as $q_{r}=k_{1r}{}^{eff}\prod_{i=1}^{ns}C_{i}^{\nu_{ir}^{\prime }}-k_{2r}{}^{eff}\prod_{i=1}^{ns}C_{i}^{\nu_{ir}^{\prime \prime }},r=1\ldots R$, where $k_{1r}{}^{eff}$, $k_{2r}{}^{eff}$ are the forward and reverse effective rate constants of the *r*-th reactions and $C_{i}$ is the molar concentration of the *i*-th species. The effective rate constants $k_{1r/2r}{}^{eff}$ are calculated as $k_{1r/2r}{}^{eff}=\frac{2}{D^{2}}\int_{0}^{D}k_{1r/2r}\left(T\right)\;D\;dD$ and take into account the effects in the framework of a 1D model. Using the law of mass action, the reverse reactions are written explicitly in the forward direction. The forward rate constants $k_{1r}{}^{eff}$ for the reactions are used assuming an Arrhenius temperature dependence $k_{1r}=AT^{n}\exp(-\frac{E_{a}}{T})$, where *A* is the pre-exponential factor, *n* is the temperature exponent, and $E_{a}$ is the activation energy (K). The reverse rate constants $k_{2r}{}^{eff}$ were related to the forward ones through the equilibrium constants. The later were determined from the thermodynamic properties [55].

The behavior of the set of differential equations was investigated using the relative sensitivity and eigenvalue analyses. The relative sensitivity is defined as [55]: $S_{i,r}^{rel}\;=\;\frac{k_{r}}{c_{i}}\;\frac{\partial c_{i}}{\partial k_{r}}\;=\;\frac{\partial \ln c_{i}}{\partial \ln k_{r}}$, where *k_r_* are the rate coefficients of the elementary reactions and *c_i_* are the species concentrations. Eigenvalue analysis of the chemical reaction system is defined as in [48], i.e., the chemical reaction systems sequence can be considered to be a simple reaction $A_{1}\overset{\;k_{12}\;}{\to }A_{2}\overset{\;k_{23}\;}{\to }A_{3}\overset{\;}{\to }\ldots$. Integral reaction flow analysis is defined as the overall formation or consumption during the chemical process [55].

### 3.3. Gas Thermal Balance

The microwave power is absorbed primarily by plasma electrons, which transfer the power to heavy particles via elastic and inelastic collisions and high gas temperatures (up to 3000 K) are achieved. The gas temperature slightly decreases in the “hot” discharge zone when moving away from the launcher (along z axis shown in Figure 1) and then drops in the “mild” plasma zone (>7.5 cm). The radial temperature distribution is considered similar to the velocity distribution, i.e., parabolic with maximum in the center of the tube. It was also assumed that the main thermal losses are due to the radial heat conduction towards the wall [35,37]. Therefore, the gas thermal balance equation can be written as:

$$\frac{p_{0}}{k_{B}}v_{0}\frac{1}{T_{0}}C_{p}\frac{dT}{dz}=-\frac{4\lambda \left(T\right)}{R^{2}}\left(T-Tw\right)+\frac{\delta }{S}\frac{dP}{dz}$$

where $v_{0}$ (m s^−1^) is the initial gas velocity, *T*_0_ (*K*)-the initial gas temperature, *p*_0_ (Pa)-the gas pressure, $k_{B}$-the Boltzmann’s constant, *C_p_* = (5/2)*k_B_*-the heat capacity at constant pressure, *S*-the plasma cross-section, *P*-the absorbed microwave power; *δ* is a coefficient expressing the fraction of absorbed power from the wave that is transferred to thermal energy of the gas. Here, the electron energy balance equation is implicitly included via the coefficient *δ*. Around 10% energy losses due to radiation and losses in the dielectric are assumed.

## 4. Results and Discussion

### 4.1. Plasma Characterization

**Figure 2:**
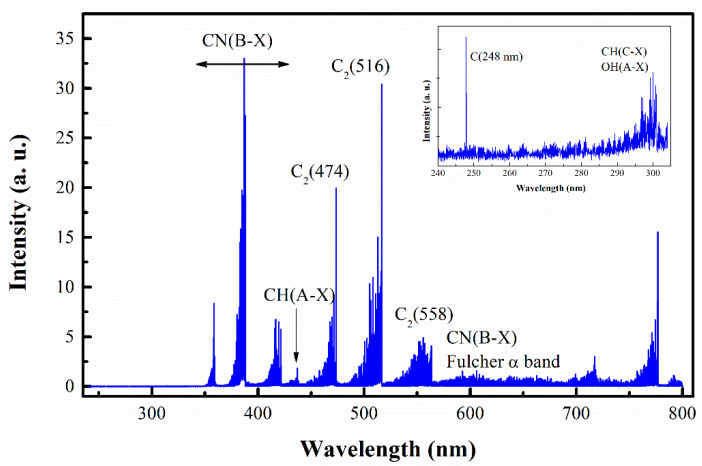
Emission spectrum from Ar/Ethanol/N_2_ plasma, at *p* = 2 kW, Q_Ar_ = 1330 sccm, Q_Et_ = 35 sccm, Q _N2_ = 10 sccm distance from the launcher z = 3 cm.

The plasma optical emission spectrum in the visible range (230–800 nm) was investigated (Figure 2). New molecular and atomic species are present in the spectra due to decomposition of the precursors in the plasma environment. The emission spectrum of argon/ethanol/nitrogen plasma is comprised of CN^•^ (B^2^Σ^+^ → X^2^Σ^+^), C_2_ (Swan system, between 450–570 nm, A^3^Πg→X’^3^Πu), CH (A^2^Δ-X^2^Π) at 431 nm, CH (C-X) at 310 nm, OH (A^2^Σ^+^→ X^2^Π_i_) and C atoms (247.9 nm). The dominant vibrational bands are C_2_ (Swan bands) and CN^•^ (B-X) violet system. The typical C_2_ emission is generated by the radiative decay of the C_2_^*^(A^3^Π_g_) state resulting in the cyan–green color of the plasma (photo in Figure 1). Due to the low energy threshold (E_ext_ = 2.4 eV), ground state C_2_ molecules can easily be excited to this level either by electron impact C_2_(X) + e → C_2_*(A^3^Π_g_) + e or by three body recombination processes involving C and Ar atoms C + C + Ar → C_2_*(A^3^Π_g_) + Ar. Additionally, atomic carbon is formed in dissociation reactions of C_2_ molecules with hydrogen radicals.

**Figure 3:**
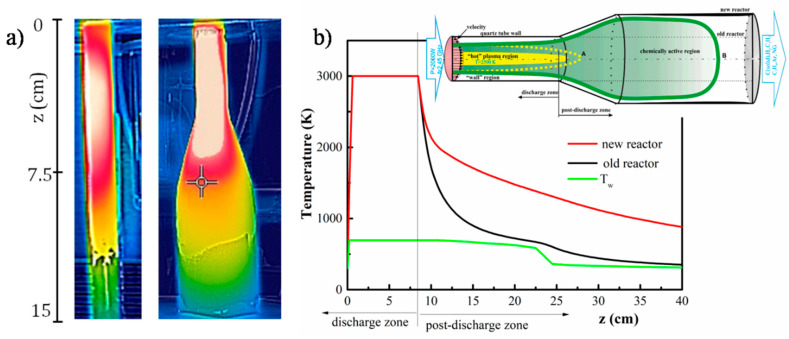
(**a**) Thermal map of the plasma–old and new reactors; (**b**) Axial temperature profile T(z) for the different reactor geometries; wall temperature profile along the tube T_w._

The gas temperature in atmospheric pressure microwave plasma is typically estimated assuming local thermodynamic equilibrium and using rotational spectra of CN^•^ or OH molecules [35,37]. Thus, the rotational temperature may be considered nearly equal to the gas kinetic temperature. Here the emission band from CN^•^ in the range 380–388.3 nm was used to estimate the rotational temperature using Lifbase software [56]. By matching the experimentally obtained rotational lines with the simulated ones a temperature of ~3000 ± 200 K was obtained. This temperature corresponds to the axial position at a distance from the launcher z = 3 cm from where the emission was collected. In fact, this is the maximum value of the gas temperature, which remains nearly constant in the plasma zone when moving away from the launcher (up to ~7 cm), establishing the so-called “hot” plasma zone and then sharply drops in the near afterglow “mild” plasma region. An indicative 2D temperature map was obtained using thermal imager (Figure 3) showing strong radial gradients of the temperature.

The process of N-graphene formation involves injection of the precursors (ethanol and N_2_) directly into the “hot” plasma zone, where their decomposition takes place. As a result of the particles collisions and multiple chemical reactions the precursors decompose down to carbon atoms and C_2_ radicals, which are the main components of the targeted materials, i.e., graphene-based structures. Additionally, nitrogen-containing species such as CN^•^, HCN NH_2_, NH_3_ are formed. The newly created species move towards colder zones (axially and radially), passing through an area with nearly constant temperature (~2500 K), the so-called “vaporization boundary” (Figure 3b green line). Here the gas-phase carbon atoms and molecules transform into solid carbon nuclei. Further clustering and coalescence of small particles into larger ones occurs in the “mild” chemically active plasma zone, during their flight with the background gas flow. A thin area near the tube wall (wall-region), with relatively low gas temperature, and where chemical reactions do not occur, can be distinguished. Some of the created particles diffuse into the wall-region and deposit on the wall. The accumulation of the particles in the chemically active zone is limited by the buffer gas flow or by their own movement (diffusion in the case of a laminar flow).

The synthesis of a specific type of structures, i.e., planar N-graphene sheets, is achieved by synergetic regulation of the “hot” plasma environment and thermodynamic conditions in the “mild” zone of the plasma reactor. Hence, by adjusting the temperature gradients, the concentration of the precursors´ fractions and their residence time in the plasma reactor, selective synthesis of graphene-like sheets in a narrow range of operational parameters can be achieved.

It should be noted that a correlation between changes in C_2_ and C concentrations in the plasma and sp^3^/sp^2^ ratio was found earlier [35]. Presence of C_2_ radicals in the plasma results in predominant assembling of sp^2^ planar structures (grapheme-like structures). Inversely, formation of more carbon atoms leads to increased synthesis of sp^3^ carbon systems [35].

The gas temperature is an essential parameter regarding plasma kinetics and is an input value in the numerical calculations. In our earlier works [35,37] a quartz reactor with constant cross-section (1.5 cm inner and 1.8 cm outer diameters) was used. In the present work, the reactor includes a section with expanding radius (Figure 1). The new geometry allows increasing the production yield while keeping the high quality of the graphene structures unchanged. For the two different quartz tube designs the geometrical dimensions of the chemically active “mild” zone, as well as the temperature profile, will be different. Significant increase of the chemically active zone occurs when the tube cross-section is expanded (from 1.5 cm to 4.2 cm), since the velocity of the heat conduction decreases. At the same time, the dimensions of the “hot” plasma zone remain almost unchanged (Figure 3).

**Figure 4:**
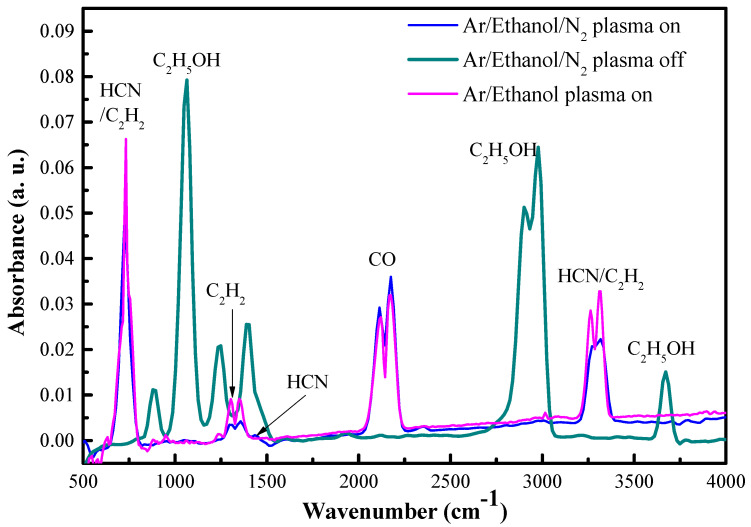
FT-IR spectra for the Ar/C_2_H_5_OH/N_2_ outlet gas with and without plasma, and the spectrum of Ar/C_2_H_5_OH with the plasma on.

The outlet gas stream from Ar/Ethanol/N_2_ plasma was investigated using FT-IR spectroscopy. The spectra detected with the plasma on and off, at typical operational parameters for N-graphene synthesis (*p* = 2 kW, Q_Ar_ = 1330 sccm, Q_Et_ = 35 sccm, Q_N2_ = 10 sccm), are shown in Figure 4. The absorption spectrum detected with plasma on, shows spectral lines attributed to CO, C_2_H_2_, and HCN. C≡O has a major absorption band at 2100 cm^−1^, due to asymmetrical vibration modes. The bands in the C_2_H_2_ spectrum can be assigned to –C≡C– stretching at 2260–2100 cm^−1^; –C≡C–H: C–H stretching at 3330–3270 cm^−1^; –C≡C–H: C–H bending at 700–610 cm^−1^. HCN normal vibration modes such as ν_2_ (a doubly degenerate bending) at 712.1 cm^−1^, ν_3_ (C-H stretching band) at 3330 cm^−1^ were observed. The features at 1412 cm^−1^ and 2117 cm^−1^ are the 2ν_2_ and 3ν_2_ overtones of the ν_2_ fundamental mode. The absorption spectrum of the Ar/Ethanol/N_2_ outlet gas under the same conditions but without plasma exhibits the peaks characteristic for ethanol molecules, associated with both the O-H at 3200–3500 cm^−1^ and the C–O stretching vibrations at 1050–1260 cm^−1^ [57,58]. The plasma completely decomposes the ethanol into the main by-products H_2_ and CO [41,42]. Additionally, the spectra of Ar/Ethanol plasma output gas (*p* = 2 kW, Q_Ar_ = 1330 sccm, Q_Et_ = 35 sccm) shows increase in the relative intensity of acetylene bands.

### 4.2. Theoretical Results

**Figure 5:**
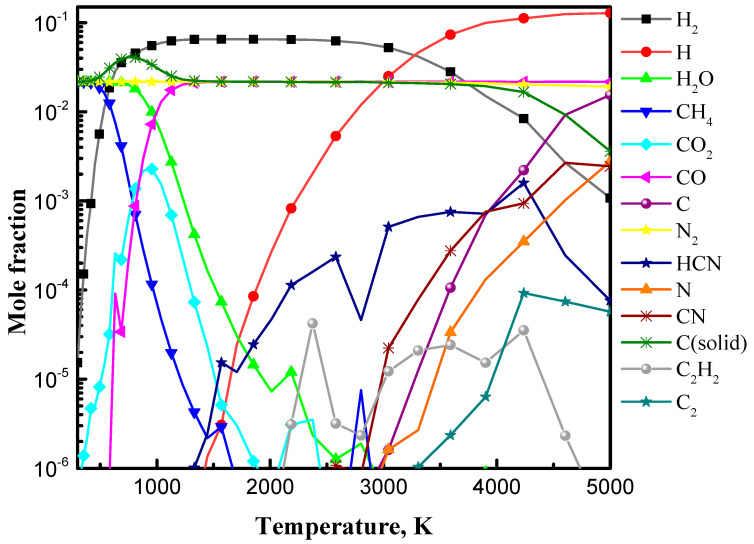
Simplified equilibrium diagram of the main decomposition products for the Ar/C_2_H_5_OH/N_2_ = 1320/30/30 mixture.

The calculated concentrations of the main decomposition products obtained under thermal equilibrium assumption are shown in Figure 5 as a function of the gas temperature. The results are acquired considering heterogeneous approximation, i.e., formation of solid-phase carbon. By comparing the equilibrium diagrams in the case of Ar/C_2_H_5_OH/N_2_ and Ar/C_2_H_5_OH a substantial difference is not observed, which implies that the presence of nitrogen does not interfere with the basic principles of ethanol decomposition established earlier [35,40]. Four characteristic temperature regions can be delimited depending on the formed stable substances and intermediate complexes. The first region is characterized by the presence of methane (up to about 1000 K); in the second region there is formation of solid-phase carbon in equilibrium state (from 1000 K to 2000 K); in the third one there is production of acetylene (from 2000 K to 3500 K); and, finally, the region of complete decomposition of C_2_ and H_2_ to gas-phase carbon and atomic hydrogen (for temperatures above 3500 K). No fundamental difference was found in the list of species and concentrations of C_x_H_y_O_z_ by comparison with [35,40].

The representatives of carbon–nitrogen species in the plasma are HCN and CN: HCN forms in the temperature region 2000 to 4000 K, while CN^•^ appears at temperature > 4000 K. Naturally at temperatures above 4000 K, hydrogen is detached from C–H as a more fragile bond compared to C=N shifting towards CN^•^ formation. The concentration of atomic carbon and nitrogen is significant above 3200 and 3700 K, respectively [59].

**Figure 6:**
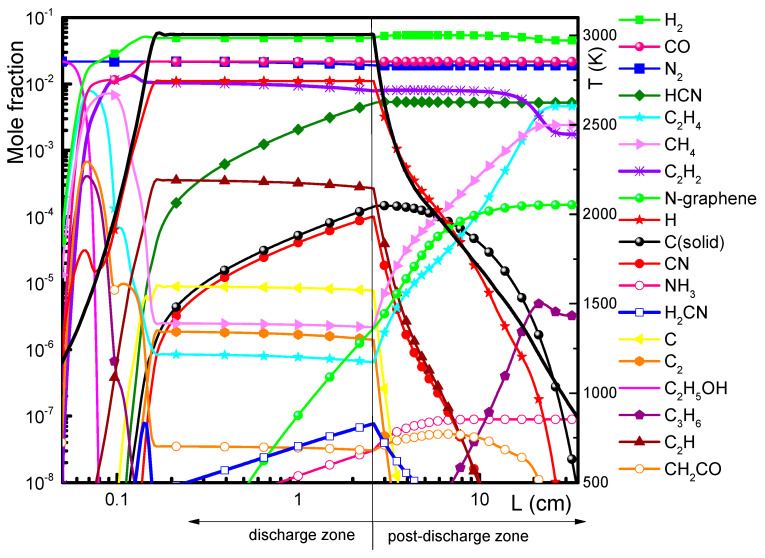
Evolution of main species concentrations along the discharge and the post-discharge zone. Ar/C_2_H_5_OH/N_3_ = 1320/30/30, *p* = 2 kW.

The results of the non-equilibrium modeling, i.e., the axial variations of the concentrations of the main decomposition products considering ethanol/nitrogen precursors, are presented in Figure 6. The calculated gas temperature profile, taking into account both the upper level of the measured gas temperature (~3000 K) and the measured wall temperature axial profile is also shown in Figure 6. As expected, the ethanol molecules decompose forming stable molecules, such as CO, C_2_, C, C_2_H_2_, C_2_H, C_2_H_4_, CH_4_, C_3_H_6_ etc., while N_2_ does not dissociate directly. Above 1500 K, the chemical reactions rates increase significantly, so the time to reach equilibrium is reduced to a few microseconds and the assumption of local thermal equilibrium is valid. At this conditions radicals and species containing nitrogen, i.e., NH_2_, NH_3_, CN^•^, HCN, N, etc., start to form. These reactions occur in the chemically active zone, which is enhanced in the new reactor geometry, therefore benefiting the formation of N-graphene. For this reason, in the following experiments the N-graphene synthesis was performed using the new reactor featuring the expanding radius geometry.

**Figure 7:**
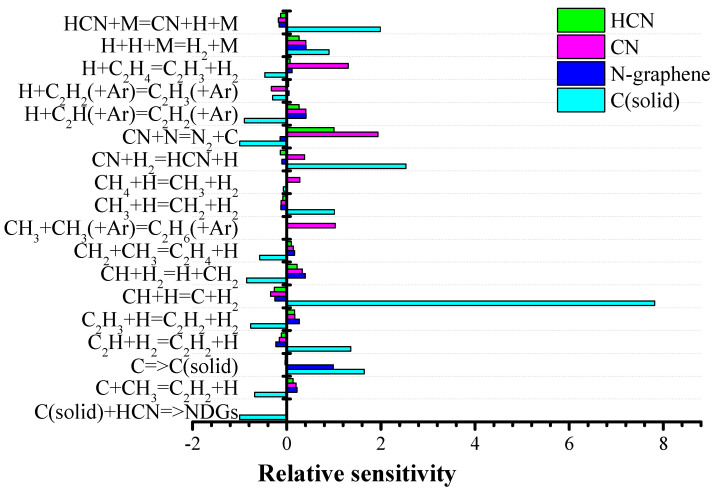
Sensitivity analysis for HCN, CN^•^, N-graphene, and C(solid) concentrations in the chemically active zone (z = 15 cm) for Ar/C_2_H_5_OH/N_2_ = 1320/30/30. (M is a third body).

**Figure 8:**
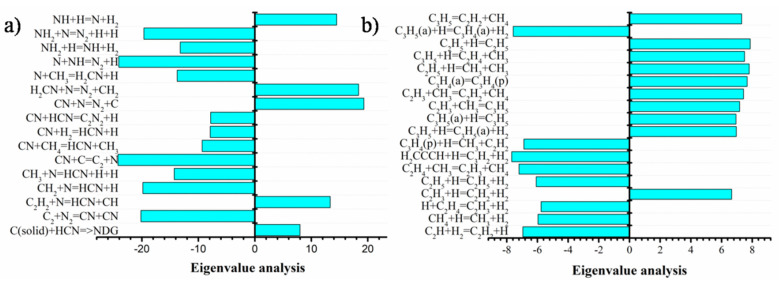
Eigenvalue analysis of (**a**) nitrogen-containing chemical reactions; (**b**) carbon-containing chemical reactions in the chemically active region (z = 15 cm) for Ar/C_2_H_5_OH/N_2_ = 1320/30/30.

Sensitivity analysis was performed to evaluate the relative influence of the different chemical reactions to the formation of HCN, CN^•^, N-graphene, and C(solid) (Figure 7). Additionally, the eigenvalue analysis considering reactions with carbon and nitrogen was carried out (Figure 8).

**Figure 9:**
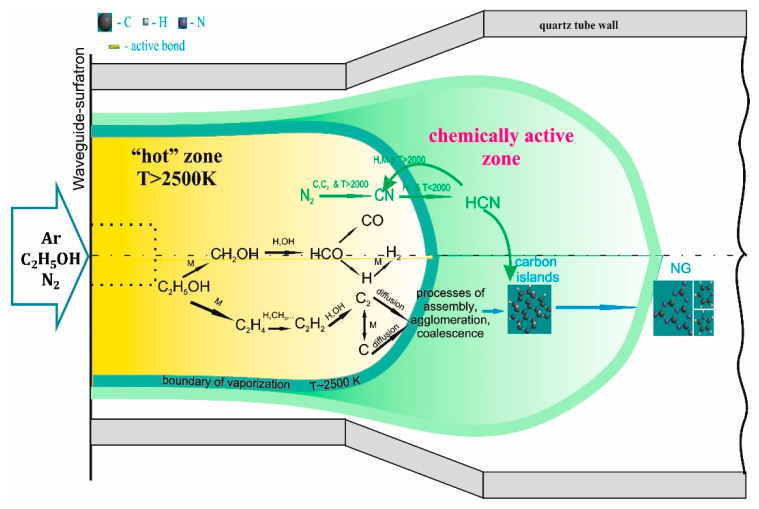
Integral reaction flow analysis of ethanol deposition and nitrogen-related chemical reactions in the plasma.

The simulation results, the sensitivity analysis and the integral reaction analyses of the kinetic mechanism in the “hot” plasma region indicate that the ethanol decomposition occurs through two parallel, approximately equivalent mechanisms (Figure 9), as previously stated [35,37,43,44]. The most important chemical reactions that affect the relative amount of H_2_ also affect the relative amount of solid carbon. The absolute difference between H_2_ and C(solid) sensitivity coefficients is due to the fact that part of the hydrogen is formed by the first mechanism, i.e., breaking the C-C bond, leading to formation of syngas (H_2_ and CO) and is, therefore, independent of the carbon diffusion rate. On the other hand, the solid carbon is only created by the second decomposition mechanism, i.e., OH group is detached from the ethanol molecule forming, after multiple reactions, C, C_2_, and H. The solid carbon formation is not related to the CO generation. CO is only created through the first mechanism and does not depend on the carbon diffusion rate.

The nitrogen molecule does not dissociate directly; the formation of nitrogen-carbon species occurs through reaction with C and C_2_, and partially, through interactions with radicals, such as CH_2_, CH etc. In this case, the formation of CN^•^ is dominant:

N_2_ + C → CN^•^ + N (R1)

C_2_ + N_2_ → CN^•^ + CN (R2)

At the same time, the CN radical is not directly involved in the formation of nitrogen-doped graphene structures. In the chemically active region, a change of mechanisms occurs:

CN^•^ + H_2_ → HCN + H (R3)

N_2_ + CH_2_ → H_2_CN + N (R4)

N_2_ + CH → HCN + N (R5)

leading to HCN generation, which is actively involved in the formation of nitrogen-doped graphene structures.

The free nitrogen atom, generated in the reaction (R1, R4, R5) mainly bonds with hydrogen and is involved in the formation of another by-product, i.e., ammonia:

N + H_2_ → NH + H, NH + H_2_ → NH_2_ + H, NH_2_ + H_2_O → NH_3_ + OH.

Since N-graphene formation is closely related with the presence of HCN, optimal temperature for this process is around 3000–4000 K, when HCN concentration reaches maximum values (see Figure 5).

Some discrepancy between the theoretical predictions and the experimental FT-IR observations were observed, with the FT-IR spectrum of the plasma outlet gas-phase products showing relatively small peak of HCN while the model predicts a significant formation of HCN (Figure 4 and Figure 6). This discrepancy can be related to unaccounted processes in the C/N/O mechanism, as well as to physico-chemical properties of HCN not considered in the model. For instance, a possible reaction, not considered in the kinetic mechanism of Klaus and Warnatz [47,48] is of C_2_H_2_ and HCN leading to formation of acrylonitrile CH_2_=CH–C≡N [60]. An indication of the possibility of this reaction taking place is the decreased concentration of the C_2_H_2_ in the FT-IR spectrum of Ar/C_2_H_5_OH/N_2_ in respect to the one in Ar/C_2_H_5_OH plasma output gas (see Figure 4). A decrease in the intensity of CH≡CH in Ar/C_2_H_5_OH/N_2_ FT-IR spectrum predicts a decrease in the concentration of CH≡CH. The concentration of CH≡CH is directly related to the concentration of CH_2_=CH_2_: CH≡CH + H_2_ ↔ CH_2_=CH_2_. Accordingly, if the concentration of CH_2_=CH_2_ decreases, then the concentration of CH≡CH decreases.

Another source for the mismatch could arise from the fact that the FT-IR measurements are performed at temperature ~22–23 °C, which is lower than the HCN boiling point 25.6 °C. Therefore, under the conditions considered the HCN is in the gas–liquid phase equilibrium and the measured signal, being proportional to the gas-phase concentration, corresponds to the HCN vapor pressure. Thus, the FT-IR signal is saturated and does not correspond to the actual HCN concentration.

**Figure 10:**
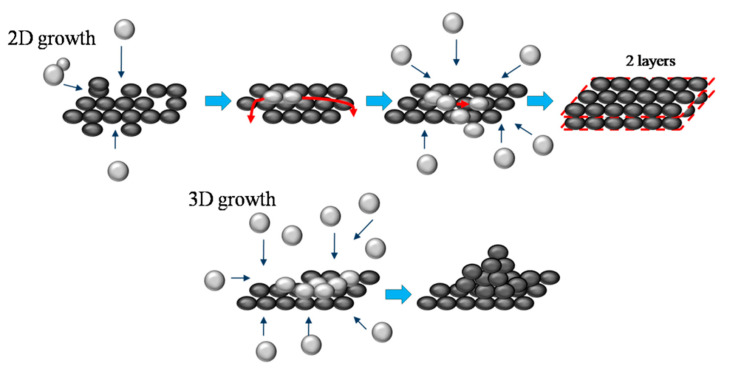
Proposed growth mechanism of free-standing 2D and 3D structures. Gray spheres represent gas-phase carbon-based species (C, C_2_, C_2_H, CH); dark spheres represent nucleation centers.

A possible mechanism for the formation of free-standing graphene-like structure is proposed. The first step is the transformation of gas-phase carbon-based species (C, C_2_, C_2_H, CH) into nucleation centers that can either grow as 2D or 3D structures, depending on the thermodynamic conditions and the concentrations of carbon species (Figure 10). When a graphene nucleation center is created it can accomplish straight forward 2D (planar) growth via attachment of carbon-based species to the edges of the nucleation center. Alternatively, the building blocks of graphene can land on the surface of the graphene sheet, where they are weakly bonded and therefore highly mobile. This may result in their fast migration along the surface until they reach the edge, where they bond and contribute to the further growth of the graphene sheet. However, these species can also encounter other building blocks already present at the surface before they reach the edge. If so, this can lead to growth of a new graphene layer on the top, i.e., to the formation of multilayer graphene. The probability of such event will increase with the area of the graphene sheet (which increases the time necessary to reach the edge of the sheet) and the concentration of the building blocks at the surface of the sheet. Therefore, the size of single layer flakes will be limited by the concentration of carbon-based species in the plasma and their diffusion rate on the sheet. If the carbon-based species concentration is further increased (i.e., the so-called supersaturation condition is achieved), larger number of nucleation centers will be created along with lower gas temperature. These conditions favor the formation of 3D structures, which would not survive at higher temperature due to their lower stability as compared to the graphene phase [37].

Hydrogen atoms from the original molecule can migrate on cluster´s surface, and form H_2_ molecules that evaporate from the surface. HCN, CN^•^ and N may attach to the graphene-like structures at the edges but also can occupy vacant defects of the graphene lattice.

The description of the actual growth process of the free-standing graphene/N-graphene cannot be achieved in the frames of the current Equilibrium and Rate balance equation approaches, but requires complex ab initio quantum methods. These notoriously complex models will be addressed in the next stage of development of the theory for the formation of the free-standing graphene-like structures. At this stage however, the description of the substrate-free graphene/N-graphene formation is limited to equilibrium and non-equilibrium approaches. For the purpose, several simplified equations are included in the non-equilibrium model, to provide quantitative estimation of the process. The formation of graphene was estimated by the rate of formation of C(solid), already existing in the heterogeneous chemical kinetics model. We assume that formation of N-graphene is governed by the reactions: C(solid) + HCN = N-graphene + 1/2H_2_, C(solid) + CN^•^ = N-graphene and C(solid) + N = N-graphene and that the rate of formation of N-graphene is limited by the rate of formation of C(solid).

### 4.3. Material Characterization

**Figure 11:**
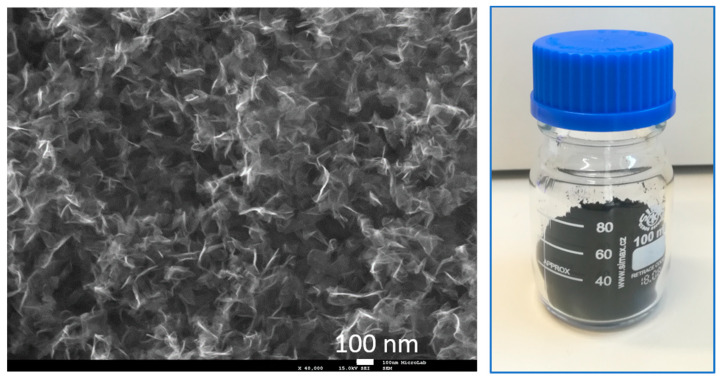
SEM image and photo of the free-standing N-graphene as synthesized at Q_Ar_ = 1320 sccm; Q_Et_ = 30 sccm; Q_N2_ = 30 sccm; *p* = 2 kW (NG1).

A SEM image of the N-graphene is shown in Figure 11, together with a photograph of the structures as synthesized at Q_Ar_ = 1320 sccm; Q_Et_ = 30 sccm; Q_N2_ = 30 sccm; *p* = 2 kW. Sheets are the only allotrope present in the sample; in this sense, the presented SEM image is representative for all the samples. It reveals the characteristic free-standing graphene morphology, consisting of curved ultrafine sheets [36,37,38,40]. The synthesized structures are substrate-free sheets, which are randomly oriented.

**Figure 12:**
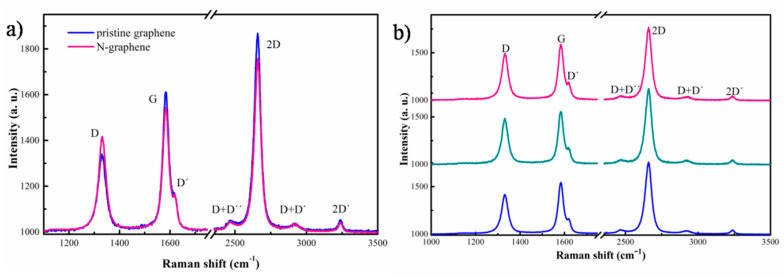
(**a**) Averaged Raman spectra of pure graphene and N-graphene; (**b**) Raman spectra at three randomly chosen locations of N-graphene sample at Q_Ar_ = 1320 sccm; Q_Et_ = 30 sccm; Q _N2_ = 30 sccm; *p* = 2 kW.

The Raman spectra of the synthesized N-graphene and pure (undoped) graphene are compared in Figure 12a. The presented spectra are the resulting averages of spectra collected over random sample locations. The pure graphene was synthesized using microwave plasma, the method is described elsewhere [32,33,34,35]. Both spectra consist of three dominant peaks at ~1331, 1582 and 2658 cm^−1^ attributed to D, G, and 2D bonds, respectively. The G peak is typical for all sp^2^ carbon systems, representing the in-plane vibrations. The position of D and D´ peaks depends on the excitation photon energy. Their intensity depends on the type of the defects and their concentration in the layer as well as on the size of graphene flakes. The 2D peak corresponds to the overtone of the D band and its intensity and shape is very sensitive to number and the stacking order of the graphene sheets [61]. As it can be seen, in the spectrum of N-graphene the defect-related D peak slightly increases in respect to the one of pure graphene. The nitrogen atoms incorporated in the graphene lattice represent defects, and consequently contribute to the D band intensity, yielding the observed increase of the D to G-band intensity ratio—from 0.55 for pure graphene to 0.76 for N-graphene. The full width at half maximum (FWHM) of 2D peak is ~52 cm^−1^ for both pure graphene and N-graphene suggesting that these structures are predominantly composed of several-layers [36,37,38]. The hypothesis for one-layer graphene also cannot be excluded as the 2D peak has Lorentzian shape and the relatively large FWHM (compared to the one observed in single layer graphene deposited on substrate) can be explained with the high concentration of linear 1D defects (foldings of the free nanosheets) [61]. Raman spectra from three different randomly chosen points of the same sample are shown in Figure 12b. The sample appears to be very homogeneous-the spectra collected from different spots on the sample are very similar. Having in mind that the interlayer distance in the graphene sheets is 3.35 Å the thickness of these structures is about 1–3 nm.

Analogous results are reported in [62], were substrate-free graphene flakes are synthesized in arc plasma using different precursors and appropriate process parameters. Dependences of the plasma buffer gas, type of the carbon-containing precursor and the arc current on the morphology of the created structures are established. It is interesting to mention that 2D to G intensity line ratio in the present spectra is higher than the one reported in [62,63] of samples synthesized in arc plasma. However, it is unclear if this is a sign for some “mildness” of the conditions of the microwave plasma synthesis compared to the arc plasma synthesis—there are too many parameters that can influence this ratio such as nitrogen content, number of layers in the graphene flake, flake size, etc. We suggest that the concentration of the foldings of the free nanosheets can also strongly influence this ratio [59].

**Figure 13:**
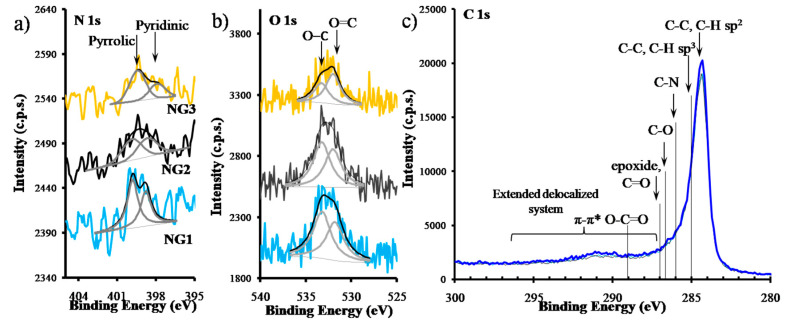
XPS regions (**a**) N 1s, (**b**) O 1s, and (**c**) C 1s from samples NG1, NG2, and NG3. N 1s regions were smoothed (average of 3 points).

Varying the precursors’ flow ratios three different N-graphene samples were synthesized, i.e., NG1, NG2, and NG3 with Q_Et_/Q_N2_ = 30/30; 30/50; 35/10, respectively (Table 1). The XPS spectra revealed that all samples are doped with nitrogen (Figure 13a). The atomic ratio N/C is the largest for the NG1 sample. However, this sample is also the one presenting the largest relative amount of oxygen. The relative amount of oxidized species is very similar for NG1 and NG2, while being considerably smaller for NG3.

Reduced amount of N-doping at the highest N_2_ flow is rather surprising. However, one should have in mind that adding nitrogen affects the thermodynamic conditions in the assembly zone of the plasma. Specifically, the presence of nitrogen increases the gas temperature. Under such conditions, the attachment of HCN species to the graphene nucleation centers becomes less probable i.e., HCN tends to stay in the gas phase and leaves the assembly zone with the exhaust gas.

In the N 1s regions (Figure 13a) it is possible to fit two peaks assigned to pyridinic and pyrrolic nitrogen, centered, approximately, at 398.4 eV and 399.7 eV, respectively. No quaternary nitrogen was detected. O 1s regions were fitted with a minimum of two peaks, assigned to oxygen in ester groups: peaks centered at 532.0 ± 0.1 eV and 533.2 ± 0.1 eV correspond to ***O***=C–O and O=C–***O***, respectively (Figure 13b). Given the difference in the peak areas, other species have to be considered: in samples NG1 and NG2 the peak at higher binding energies (BE) can also include oxygen from epoxide groups, typically centered at 533.15 eV [64] in sample NG3 the peak at lower BE can have the contribution of oxygen from carbonyl groups, usually detected between 531.3 and 532.3 eV, depending on the vicinity [64]. In fact, all regions have a small shoulder, barely seen, at the low BE side, close to 531 eV, assigned to C=O bonded to aromatic rings [64]. C 1s regions show the two main features typical of an aromatic system: the main peak centered at 284.4 eV attributed to sp^2^ carbon atoms and the region roughly between 287 eV and 295 eV corresponding to energy losses due to π-π* electronic excitations (Figure 13c). The typical positions of the other carbonaceous species are indicated in the spectrum. The oxidized carbon is in a residual amount, as attested by the atomic concentration of oxygen (Table 1). The main contribution to the C 1s peak asymmetry is from the sp^3^ contribution and the corresponding vibronic fine structure which extends to the high BE side of the main peak.

As predicted by the model, the production yield of N-graphene was small (<0.5 mg/min), with part of nitrogen apparently being lost as gas-phase N_2_, CN^•^ and HCN, while conversion to CO is the main carbon loss mechanism. Due to the high stability of N_2_, direct dissociation of the molecule practically does not occur.

## 5. Conclusions

In this study, experimental and theoretical investigations of a microwave plasma-based direct synthesis of free-standing N-graphene sheets using ethanol and nitrogen as precursors are presented. The work is a continuation of a series of investigations related to plasma-enabled synthesis of advanced 2D nanostructures. The updated theoretical model, considering the nitrogen precursor, is based on plasma thermodynamics and chemical kinetics mechanism of ethanol decomposition. The main results can be summarized as follows:

- The presence of nitrogen in the plasma environment does not change the main mechanism of ethanol decomposition, as previously investigated.
- The nitrogen molecules do not dissociate directly. The formation of carbon–nitrogen species mainly occurs in reactions with gaseous atomic and molecular carbon. The CN^•^ radical does not directly participate in the formation of nitrogen-doped graphene structures.
- The process of HCN attachment to the graphene-like structures which takes place in the chemically active region is the main mechanism of N-graphene formation.
- The new reactor geometry, with increased cross-section, was found to influence the nitrogen doping, since it provides an enhanced chemically active zone where the nitrogen-containing species form and attach to the graphene structure.
- The theoretical predictions of the updated model are in a good agreement with all experimental data and can assist in deeper understanding of the complicated physical and chemical process in microwave plasma.
- Free-standing N-graphene sheets with high structural quality as demonstrated by Raman and SEM analysis were synthesized.
- Using N_2_ as precursor, relatively low doping levels, with nitrogen in pyridinic/pyrrolic configuration, as attested by XPS, and low production yields (0.1 mg/min) were achieved.

## Figures and Tables

**Table 1:** Atomic concentrations (%) and atomic ratios.

Q_Ar_ (sccm)	1320	1380	1330
Q_Et_ (sccm)	30	30	35
Q_N2_ (sccm)	30	50	10
Q_Et_/Q_N2_	1	0.6	3.5
**Sample**	**NG1**	**NG2**	**NG3**
***XPS At. Conc. (%)***			
**C**	96.9	97.2	98.6
**O**	2.8	2.7	1.2
**N**	0.24	0.16	0.14
***Ratios***			
**N/C**	0.0025	0.0017	0.0014
**O/C**	0.030	0.028	0.012
